# Myasthenia gravis associated with invasive malignant thymoma: two case reports and a review of the literature

**DOI:** 10.1186/1752-1947-8-340

**Published:** 2014-10-13

**Authors:** Said R Beydoun, Hui Gong, Nazely Ashikian, Richard Alan Rison

**Affiliations:** 1 Department of Neurology, University of Southern California, Keck School of Medicine, Los Angeles County Medical Center, 1520 San Pablo Street, Los Angeles, CA 90033, USA; 2 Department of Neurology, Kaiser Permanente, 15788 Midwood Drive, Granada Hills, CA 91344, USA; 3 Department of Neurology, PIH Health Stroke Program, 12401 Washington Boulevard, Whittier, CA 90602, USA

**Keywords:** Thymomatous myasthenia gravis, Invasive malignant thymoma, Thymoma, Myasthenia gravis, Plasmapheresis, Intravenous immunoglobulin

## Abstract

**Introduction:**

Approximately ten to fifteen percent of patients with myasthenia gravis are found to have a thymoma, and twenty to twenty-five percent of patients with thymoma have myasthenia gravis. Thymomatous myasthenia gravis tends to have a difficult clinical course and poor prognosis.

**Case presentation:**

We report two cases (one patient of Asian ethnicity and the other of Caucasian ethnicity) of atypical presentations of myasthenia gravis associated with invasive malignant thymoma. Both patients were diagnosed at a young age, in their 20s. They presented with a turbulent course of myasthenia gravis and recurrent thymoma, but obtained good outcome after aggressive treatment involving multiple different specialists.

**Conclusions:**

Although thymomatous myasthenia gravis tends to have a difficult clinical course and poor prognosis, early and aggressive treatment along with multidisciplinary management may improve the outcome of these patients.

## Introduction

Although the relationship between myasthenia gravis (MG) and thymoma is well documented, the prevalence and characteristics of MG associated with invasive thymoma are less known. Approximately ten to fifteen percent of patients with MG are found to have a thymoma. Conversely, in patients with thymoma, 40 percent have an associated autoimmune condition, presumably paraneoplastic in etiology. Twenty to twenty-five percent of patients with thymoma have myasthenia gravis [1].

Thymoma-associated MG (T-MG) is identified as a tumor originating from thymic epithelial cells, most being cortical subtype (World Health Organization type B) [2]. Because cortical thymoma usually has some morphological similarities with the thymic cortex, they share the ability to propagate the maturation of immature naive CD4 T cells and spread mature naive T cells into the periphery. The pathogenesis of T-MG suggests that epithelial neoplastic cells encircled by maturing T cells are capable of expressing epitopes cross-reactive with skeletal muscle proteins, such as acetylcholine receptor (AChR) [3]. AChR antibodies have been detected in all patients with T-MG. The AChR antibody directly attacks the neuromuscular junction, affecting the endplate region of the postsynaptic membrane, specifically aimed at the nicotinic acetylcholine receptors [4].

The patient’s genetic profile may also play an important role as well as the thymic ability to export autoreactive T cells in developing MG. Research has shown that MG has a genetic association to HLA-DR3 or ancestral haplotype 8.1 in early-onset MG. The thymoma patients with a particular genetic profile convey high risk of developing MG [5].

The incidence of thymoma in the United States is 0.15 per 100,000 person-years. Asians, Pacific Islanders and African Americans appear more frequently among thymoma patients [6]. T-MG is equally common in men and women and occurs at any age with a peak onset around 50 years [3]. Thymoma is a unique thymic epithelial neoplasm with indolent growth and rarely presents with local invasiveness and metastases [6].

Thymoma is graded according to the World Health Organization (WHO) histological classification [7] and is classified by the Masaoka staging system [8]. Above stage II is considered to be invasive. The incidence of MG associated with invasive high-stage malignant thymoma is rare. The severity of the MG is evaluated according to the Medical Scientific Advisory Board of the Myasthenia Gravis Foundation of America clinical classification system (MGFA), which was established in 2000 [9].

Thymomatous MG tends to have a difficult clinical course and poor prognosis [10]. We report here two cases of atypical presentations of MG associated with invasive malignant thymoma. Both patients were diagnosed at a young age, in their 20s. They presented with a turbulent course of MG and recurrent thymoma, but obtained good outcome after aggressive treatment.

## Case presentation

### Case 1

A 25-year-old, right-handed, Caucasian woman noticed the inability to perform certain yoga positions with fatigability and decreased stamina for exercise beginning at the age of 22. She soon developed diplopia, facial and proximal muscle weakness with difficulty lifting her arms above her head and difficulty climbing stairs. She was admitted to a community hospital and had an extensive workup, including brain and spine images as well as serum autoimmune studies, which were normal. She was given one cycle of intravenous immunoglobulin (IVIG), which slightly improved her symptoms.

Her condition deteriorated with difficulty chewing, swallowing, and increasing weakness. She was referred to us and manifested classical significant myasthenic weakness in her extraocular, bulbar and proximal arm and leg muscles. Repetitive nerve stimulation studies revealed 17 percent compound muscle action potential (CMAP) amplitude decrement in the right median nerve and 19 percent in the left spinal accessory nerve. Forced vital capacity was 1.65 liters. Her AChR binding antibody was elevated at 133.06 and blocking antibody was positive at 43. She was classified as MGFA class IVb. Prednisone, IVIG, and pyridostigmine were initiated.

A chest computed tomography (CT) scan showed a large anterior mediastinal heterogeneous mass with solid and cystic components measuring 6cm × 4.6cm × 5.2cm with invasion of the pericardium. Video-assisted thoracoscopy (VATS) and anterior mediastinoscopy were performed. An extensive lesion was noted with local adherence to the pulmonary left upper lobe and presence of a left pleural effusion. The invasive thymoma was classified as Masaoka stage III.

She received neoadjuvant chemotherapy consisting of cisplatin and etoposide with 45Gy of radiation for tumor reduction. She had a complicated clinical course with numerous myasthenia crises requiring repeated intensive care admission and endotracheal intubation for ventilator support. Plasmapheresis and IVIG were administered at different times. She later underwent a median sternotomy for a radical thymectomy with *en bloc* resection of a portion of the left upper lobe of the lung. The final pathology showed WHO type B1 with less than 10 percent residual viable tumor. Five months after the initial tumor was biopsied, she was found to have recurrent focus of tumor on the left inferior phrenic nerve margin requiring adjuvant radiation for another six weeks.

Serial chest imaging showed no evidence of recurrent tumor. She is currently two years post thymectomy and has significantly improved to an MGFA minimal manifestation status (MMS). She is currently maintained on a low dose of both prednisone and pyridostigmine without IVIG.

### Case 2

A 25-year-old, right-handed, Asian man started to notice difficulty in chewing and weakness in both arms and legs at age 20. He was given prednisone at a community hospital but his symptoms did not improve. When he was referred to us one month later, he had quickly progressed to an inability to ambulate, dysphagia, slurred speech, severe ptosis, weakness chewing and diplopia. His AChR-binding antibody was positive at 30.38 and blocking antibody was elevated at 36. Forced vital capacity was 1.3 liters. He was given IVIG, but no definite improvement was noted. He was admitted to the intensive care unit in myasthenia crisis and given aggressive plasmapheresis and immunotherapy. He was classified as generalized myasthenia gravis MGFA class IVb.

A CT scan of his chest revealed a 7.6cm × 6.1cm anterior mediastinal mass with local invasion as well as some pleural nodules, the largest of which was 2.4cm × 1.1cm. A biopsy was done through VATS and his invasive thymoma was classified as Masaoka stage IVa with pleura dissemination. He was started on neoadjuvant chemotherapy consisting of cisplatin and etoposide with radiation.

A turbulent clinical course ensued requiring repeated plasmapheresis and IVIG. Our patient then improved. His repeat chest image showed significant decrease in the size of the anterior mediastinal mass and resolution of the posterior pleural thickening five months later. He was taken to the operating room (OR) and underwent median sternotomy with resection of the anterior mediastinal mass, total radical thymectomy and resection of the innominate vein successfully. Radiation therapy continued postoperatively. The final pathology showed WHO type AB thymoma with invasion to the pleura and blood vessel wall of the innominate vein.

Fluctuation and a difficult course followed requiring a combined therapy including prednisone, pyridostigmine, IVIG and azathioprine. He slowly improved to MGFA class IIa and his prednisone and IVIG were gradually reduced four months after the operation.

Serial chest imaging was performed and a pulmonary cryptococcal nodule was detected two years following the initial diagnosis of MG, which prompted fluconazole treatment. Three years after the diagnosis, he reached MGFA MMS and was maintained on low-dose prednisone and azathioprine.

Our patient’s MG relapsed again after two years with slurred speech, ptosis, double vision, and extremity weakness secondary to medication noncompliance. He was restarted immediately on prednisone, azathioprine and IVIG; chest imaging showed increased size of a left posteromedial pleural nodule to 2.7cm × 0.9cm. A biopsy showed recurrent metastatic thymoma.

Chemotherapy with cisplatin, cytoxan, and doxorubicin was begun. He underwent recurrent thymoma resection five months later with left posterolateral thoracotomy with excision of two pleural masses and excision of the old lesions of the diaphragm with primary repair. The final pathology showed metastatic thymoma in the left pleura and diaphragm. He improved gradually after thoracotomy. He is currently MGFA Class IIa and remains on combination therapy of prednisone, azathioprine and IVIG.

## Discussion

Several retrospective studies have been conducted to identify variables that influence the natural history of T-MG and therapeutic approaches. Maggi *et al.* reviewed 197 patients with T-MG, with mean age at onset being approximately 47 years. Twelve percent of the patients had invasive thymoma in Masaoka stage III, 3 percent in stage IVa and 0.5 percent in stage IVb. Mean age at thymectomy was about 48 years. Almost 10 percent of the patients had recurrent thymoma, which was 17 percent with invasive thymoma stage III [11].

The study identified that early onset age before 45 as well as invasive thymoma were significantly associated with the persistence of symptoms. Invasive thymoma was correlated with a higher probability of recurrence. The chance of achieving remission in T-MG was 9.64 percent, but of those the majority were noninvasive stage I thymoma [11].

Other studies looked into the prognostic factors for T-MG and revealed that WHO classification had limitations in accurately predicting the prognosis of the disease [6]. Although a study led by Ruffini *et al.* demonstrated that WHO histologic classification and Masaoka stage are interrelated in T-MG, in multivariate analysis only Masaoka stage was an independent prognostic factor [12]. Furthermore, histological features had a lesser role in foreseeing outcome after resection, thus Masaoka stage is believed to be a better predictor of clinical behavior, recurrence, and long-term survival.

One study found the rate of complete resectability varied by stage; essentially 100 percent resectability for stage II, 50 percent to 60 percent for stage III, and approaching 0 percent for stage IVa tumors [13]. Only completeness of resection was significantly correlated with a better prognosis [14].

Another study demonstrated that survival rate was significantly different among stage I, stage III, and stage IVa with a significant correlation between complete resection and survival. Significant correlation was found between no invasion to the great vessel, invasion except for the great vessel, and invasion to the great vessel [15].

Invasiveness remains the best predictor of relapse-free survival after resection. Drop metastasis into ipsilateral pleura and pericardium is a characteristic finding in invasive thymoma. The metastases are frequently confined to the pleura, pericardium, or diaphragm [6].

Lucchi *et al.* reviewed 123 patients diagnosed with T-MG with a mean age of 56 years who underwent thymectomy and revealed that higher complete MG remission rate was achieved in early-stage thymoma. This is the first study documenting that T-MG patients who achieved a complete remission in MG had a significantly better prognosis. Overall survival was dependent on the Masaoka stage, the WHO classification, and the achievement of complete remission of myasthenia symptoms [16].

Compared to the above statistical data of incidence, survival rate, prognostic factors and, recurrent frequency of the T-MG, the two cases of MG associated with invasive thymoma we report here are rare.

Our patients’ symptoms of MG occurred at very young age, 20 and 22 years old respectively, whereas studies showed the mean age of onset was 47 to 56 with a peak at 50 years. Among 197 patients in the Maggi *et al.* study, only 10 patients were in the age group 20 to 29 years with low-stage invasive thymoma, and none of them were above stage III. Of patients of all ages with WHO type B, only two had Masaoka stage III. None of the patients with WHO type AB had Masaoka stage IV. The incidence of young patients with MG associated with high-stage invasive thymoma is extremity sporadic, which highlights the continued challenge in managing MG associated with malignant thymoma and the necessity of multi-specialist involvement.

Although radial surgical resection is the cornerstone of therapy in T-MG, possibility of completeness of resection is very low in high-stage invasive thymoma and almost impossible for stage IVa tumors [17]. The consensus is that patients with invasive thymoma, especially stage III or IV, should receive adjuvant radiotherapy and chemotherapy, however, to date, no standard therapeutic strategy has been validated.

Unfortunately, the recurrence of invasive thymoma is a frequent event. When the thymoma invades the pleura or the pericardium, radical excision is nearly impossible.

Studies have identified that young onset age, high-stage invasive thymoma, recurrence rate and inability to achieve complete remission in MG lead to poor prognosis for T-MG patients. Both of our patients, however, had favorable outcomes despite those factors. The reasons for our success are multifactorial including:

1. Early and accurate diagnosis to safeguard the myasthenia crisis.

2. Plasmapheresis and IVIG infusion prior to surgery to remove a great deal of the circulating surviving pathogenic antibodies.

3. Aggressive neoadjuvant chemotherapy and radiotherapy to shrink the tumor size to ensure a complete surgical resection and continuation of radiation post operation for any further invasion.

4. Adequate long-term combined immunosuppression and immunomodulation treatment to achieve control of the clinical symptoms.

5. Regular image surveillance to detect any recurrent tumor in the early stage.

Most importantly, thymomatous MG is a distinct disease, and the collaboration among thoracic surgeon, oncologist, radiologist and neurologist to monitor the neurological and oncological symptoms optimizes treatment through a synergistic effect and provides a favorable outcome to the patients.

## Conclusions

There is a known and established relationship between thymoma and myasthenia gravis. Although thymomatous myasthenia gravis tends to have a difficult clinical course and poor prognosis, early and aggressive treatment along with multidisciplinary management may improve the outcome of these patients.

## Consent

Written informed consent was obtained from both patients for publication of this case report and any accompanying images. A copy of the written consent is available for review by the Editor-in-Chief of this journal.

## Competing interests

The authors declare that they have no competing interests.

## Authors’ contributions

HG, NA and SRB were both involved in clinical diagnostic evaluation and management. HG, NA and SRB performed the electrodiagnostic studies. HG and NA generated the first draft of the manuscript. SRB and RAR reviewed the manuscript. RAR revised and edited the manuscript using an additional literature search. SRB and RAR were responsible for the intellectual content of the manuscript. All authors participated in and provided significant contributions in writing the manuscript. All authors read and approved the final manuscript.

## Authors’ information

HG is a neurophysiology fellow at the University of Southern California-Keck School of Medicine-Los Angeles County Medical Center. NA is a staff neurologist at Kaiser Permanente. SRB is a professor of neurology at the University of Southern California-Keck School of Medicine-Los Angeles County Medical Center. SRB is Director of the University of Southern California Neuromuscular Program, a Fellow of the American Academy of Neurology and the American Association of Neuromuscular and Electrodiagnostic Medicine, and is board certified by the American Board of Psychiatry and Neurology in Neurology, Clinical Neurophysiology, Pain Medicine, and Neuromuscular Medicine. SRB is also board certified by the American Board of Electrodiagnostic Medicine in Electrodiagnostic Medicine. SRB is a member of the advisory board and the scientific committee of the Myasthenia Gravis Foundation of California. RAR is a Deputy Editor for the *Journal of Medical Case Reports*, an Associate Neurology Editor for *BMC Neurology*, *Grand Rounds* and *WebmedCentral*, and a Section Editor for *BMC Research Notes*. RAR practices general neurology at Neurology Consultants Medical Group, serves as Medical Director of the PIH Health Stroke Program, is a Clinical Assistant Professor of Neurology at the University of Southern California-Keck School of Medicine-Los Angeles County Medical Center, and is a Fellow of the American Association of Neuromuscular and Electrodiagnostic Medicine. RAR is board certified by the American Board of Psychiatry and Neurology in Neurology and Vascular Neurology, and Neurocritical Care and Neuroimaging by the United Council of Neurologic Subspecialties. RAR is also board certified by the American Board of Electrodiagnostic Medicine in Electrodiagnostic Medicine. RAR is a former president of the Los Angeles Neurological Society and a current Fellow of the American Academy of Neurology.
